# *HSPA9*/mortalin inhibition disrupts erythroid maturation through a *TP53*-dependent mechanism in human CD34+ hematopoietic progenitor cells

**DOI:** 10.1016/j.cstres.2024.03.006

**Published:** 2024-03-19

**Authors:** Christopher Butler, Morgan Dunmire, Jaebok Choi, Gabor Szalai, Anissa Johnson, Wei Lei, Xin Chen, Liang Liu, Wei Li, Matthew J. Walter, Tuoen Liu

**Affiliations:** 1 Department of Biomedical Sciences, West Virginia School of Osteopathic Medicine, Lewisburg, WV, USA; 2 Department of Medicine, Division of Oncology, Washington University School of Medicine, St. Louis, MO, USA; 3 Department of Biomedical Sciences, Burrell College of Osteopathic Medicine, Las Cruces, NM, USA; 4 Department of Pharmaceutical and Graduate Life Sciences, Manchester University College of Pharmacy, Natural and Health Sciences, Fort Wayne, IN, USA; 5 Department of Pharmaceutical and Clinical Sciences, College of Pharmacy and Health Sciences, Campbell University, Buies Creek, NC, USA; 6 Department of Cancer Biology, Wake Forest School of Medicine, Winston-Salem, NC, USA; 7 Department of Biomedical Sciences, Joan C. Edwards School of Medicine, Marshall University, Huntington, WV, USA

**Keywords:** Erythroid maturation, Myelodysplastic syndrome, Del(5q), *HSPA9*/mortalin, *TP53*/p53

## Abstract

Myelodysplastic syndromes (MDS) are a heterogeneous group of clonal hematopoietic stem cell malignancies characterized by abnormal hematopoietic cell maturation, increased apoptosis of bone marrow cells, and anemia. They are the most common myeloid blood cancers in American adults. The full complement of gene mutations that contribute to the phenotypes or clinical symptoms in MDS is not fully understood. Around 10%–25% of MDS patients harbor an interstitial heterozygous deletion on the long arm of chromosome 5 [del(5q)], creating haploinsufficiency for a large set of genes, including *HSPA9*. The *HSPA9* gene encodes for the protein mortalin, a highly conserved heat shock protein predominantly localized in mitochondria. Our prior study showed that knockdown of *HSPA9* induces *TP53*-dependent apoptosis in human CD34+ hematopoietic progenitor cells. In this study, we explored the role of *HSPA9* in regulating erythroid maturation using human CD34+ cells. We inhibited the expression of *HSPA9* using gene knockdown and pharmacological inhibition and found that inhibition of *HSPA9* disrupted erythroid maturation as well as increased expression of p53 in CD34+ cells. To test whether the molecular mechanism of *HSPA9* regulating erythroid maturation is *TP53*-dependent, we knocked down *HSPA9* and *TP53* individually or in combination in human CD34+ cells. We found that the knockdown of *TP53* partially rescued the erythroid maturation defect induced by *HSPA9* knockdown, suggesting that the defect in cells with reduced *HSPA9* expression is *TP53*-dependent. Collectively, these findings indicate that reduced levels of *HSPA9* may contribute to the anemia observed in del(5q)-associated MDS patients due to the activation of *TP53*.

## Graphical abstract

**Figure:**
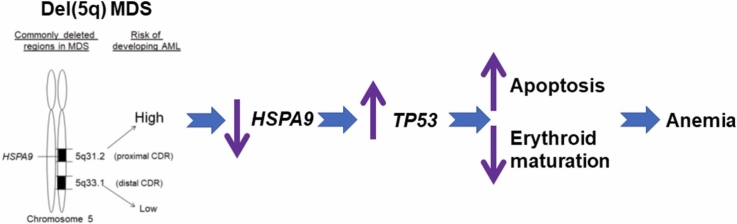

## Introduction

Heat shock proteins (HSPs) constitute a group of proteins involved in assisting the folding and maturation of other proteins, and their expression is normally induced by heat shock or other stressors. Traditionally, HSPs are known as molecular chaperones due to their physiological and protective roles in cells. They facilitate protein folding and maintenance of natural structures and functions of other proteins when cells are exposed to homeostatic challenges such as extreme temperature, anoxia, hypoxia, heavy metals, drugs, or other chemical agents that may induce stress or protein denaturation.^1^ The human HSPs are generally classified according to their molecular weights with the majority belonging to the groups HSP27, HSP40, HSP60, HSP70, HSP90, and large HSPs (HSP110 and GRP170).^2^ The human HSP70 family consists of 13 members encoded by the *HSPA* genes. HSP70 proteins have a highly conserved domain structure, including the ∼44 kDa N-terminal ATPase domain, ∼18 kDa substrate-binding domain, and ∼10 kDa C-terminal domain.^2^, ^3^ The *HSPA9* gene, encoding the protein mortalin, is located on human chromosome 5q31. The role of mortalin has been implicated in various cancer types. For example, overexpression of mortalin is detected in breast and liver cancers, and is associated with cell migration, invasiveness, epithelial-mesenchymal transition, and metastasis.^4^, ^5^, ^6^ Elevated levels of mortalin are clinically associated with poor prognosis and survival in patients with gastric and colorectal cancers.^7^, ^8^ In human colorectal adenocarcinoma cells, mortalin binds to and sequesters p53 (encoded by the *TP53* gene) in the cytoplasm, thereby preventing the translocation of p53 into the nucleus, indicating its role in regulating cell cycle and apoptosis.^9^

**Fig. 1:**
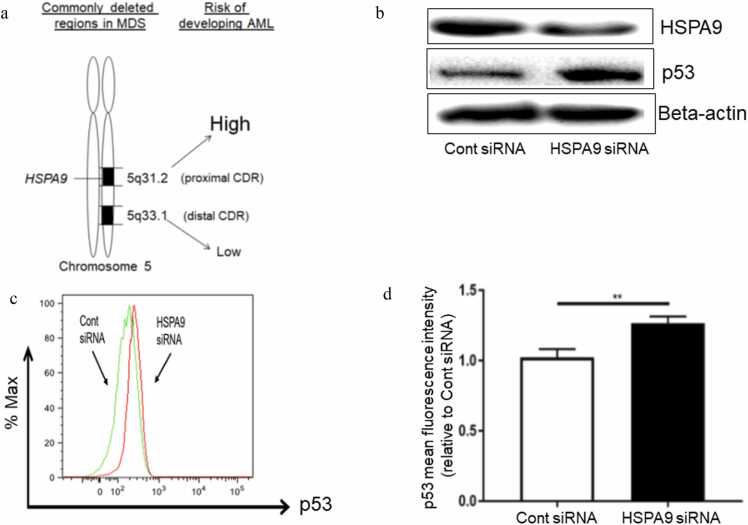
Knockdown of *HSPA9* by siRNA increases p53 expression in human CD34+ hematopoietic progenitor cells. (a) Genetic model of del(5q). Two CDRs are presented in del(5q) MDS patients. Proximal CDR contains *HSPA9* and its deletion is associated with the risk of developing AML. (b) Expression of *HSPA9*/mortalin and p53 in human CD34+ cells after siRNA transfection for 72 h measured by western blotting. Beta-actin was used as the loading control. The blots shown are a presentation of two independent experiments. (c) Flow cytometry plot of p53 in human CD34+ cells after siRNA transfection for 72 h. (d) Representative histogram of intracellular p53 levels measured by flow cytometry in human CD34+ cells following *HSPA9* knockdown by siRNA. (d) Mean fluorescence intensity of p53 following *HSPA9* knockdown by siRNA. All error bars represent SD, N = 3 technical replicates, representative of two independent experiments. **P < 0.01. Abbreviations used: AML, acute myeloid leukemia; CDR, commonly deleted regions; MDS, myelodysplastic syndromes; SD, standard deviation; siRNA, small interfering RNA.

Myelodysplastic syndromes (MDS) are a heterogeneous group of clonal hematopoietic stem cell malignancies characterized by abnormal hematopoietic cell maturation, increased apoptosis of bone marrow cells, and peripheral blood cytopenias.^10^ Patients with MDS present with symptoms of fatigue, shortness of breath, pallor, unusual bruising or bleeding, petechiae, and frequent infections.^11^, ^12^ Even with intervention and treatment, a fraction of MDS patients will progress to secondary acute myeloid leukemia, characterized by a higher percentage of blasts in either the bone marrow or peripheral blood.^13^, ^14^ Approximately 10–15% of MDS cases acquire an interstitial deletion on chromosome 5q, known as del(5q).^15^, ^16^ Del(5q) is among the most common cytogenetic aberrations in MDS and defines a unique MDS subcategory, representing the first genomic alteration included in the World Health Organization classification of MDS.^17^ Two distinct commonly deleted regions (CDR), distal and proximal CDR, have been identified on the del(5q) region in MDS patients. The *HSPA9* gene is located in the proximal CDR which is associated with patients that have a higher risk of progressing from MDS to secondary acute myeloid leukemia compared to the distal CDR (Figure 1(a)).^18^, ^19^, ^20^

In addition to its function as a molecular chaperone, the role of *HSPA9*/mortalin in hematopoiesis and erythroid maturation has been studied in nonhuman models. Zebrafish with a homozygous point mutation in *Hspa9* present phenotypically with a variety of abnormalities including severe anemia, defects in erythroid differentiation, and elevated apoptosis.^21^ Tai-Nagara *et al*.^22^ reported that inhibition of mortalin function increased reactive oxygen species and decreased the number of hematopoietic stem cells in mice, suggesting its essential role in maintaining hematological hemostasis by regulating oxidative stress. We reported that *Hspa9* heterozygous deletion mice have normal basal hematopoiesis, but display altered B-cell lymphopoiesis.^23^ This is consistent with our later reports showing that haploinsufficiency of multiple del(5q) genes, including *Hspa9*, also induces B-cell abnormalities in mice.^24^

To further examine the relationship between *HSPA9* and erythroid maturation in humans, we used human CD34+ hematopoietic progenitor cells as our experimental model in this study. We inhibited the expression of *HSPA9* in these cells using gene knockdown or an allosteric inhibitor and assessed erythroid maturation.^25^, ^26^ We have previously identified that *HSPA9* knockdown induces apoptosis in human CD34+ cells, which is likely a *TP53*-dependent process, suggesting that reduced levels of *HSPA9* may contribute to *TP53* activation and increased apoptosis observed in del(5q)-associated MDS.^27^ In this study, we measured the expression of *TP53* after *HSPA9* inhibition, in order to elucidate whether the mechanism of altered erythroid cell maturation induced by reduced *HSPA9* expression is also *TP53*-dependent.

## Results

### Knockdown of *HSPA9* by siRNA increased p53 expression in human CD34+ hematopoietic progenitor cells

To study the role of *HSPA9* in regulating erythroid maturation, we knocked down the expression of *HSPA9* in human CD34+ hematopoietic progenitor cells grown in erythroid differentiation media to explore the mechanism of anemia observed in del(5q) MDS patients. We first transfected small interfering RNA (siRNA) targeting *HSPA9* into human CD34+ cells and studied the relationship between *HSPA9* knockdown and p53 expression. We found that *HSPA9* siRNA effectively inhibited the expression of its encoding protein mortalin to ∼50%, similar to levels occurring in heterozygous del(5q) deletions in patients, and increased the expression of p53 in these cells compared to control siRNA measured by western blotting (Figure 1(b)). Flow cytometry analysis also showed that *HSPA9* knockdown resulted in increased p53 expression (Figure 1(c)) based on the mean fluorescent intensity (*P* < 0.01) (Figure 1(d)).

### Knockdown of *HSPA9* by siRNA inhibited cell growth, increased cell apoptosis, and inhibited erythroid maturation in human CD34 + hematopoietic progenitor cells

**Fig. 2:**
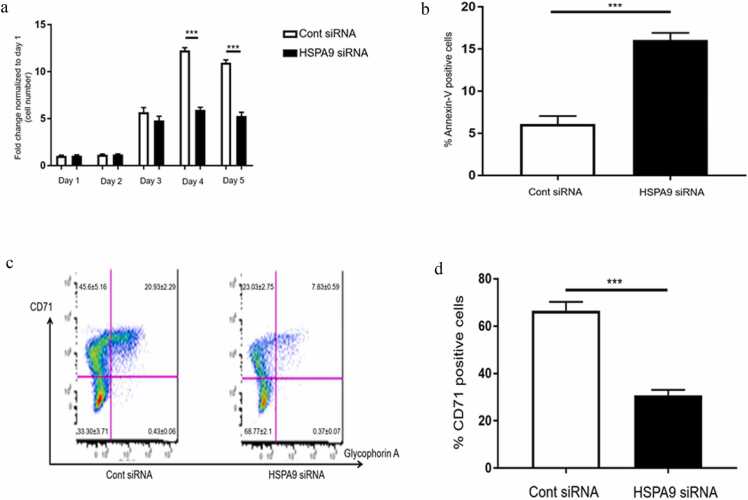
Knockdown of *HSPA9* by siRNA increases apoptosis and inhibits erythroid maturation in human CD34+ hematopoietic progenitor cells. (a) The number of human CD34+ cells was measured after siRNA transfection for 5 days. The fold change in cell counts was calculated relative to the number of cells on day 1 (N = 3 for each siRNA). (b) Quantification of annexin V+ cells, which were considered as apoptotic cells, measured by flow cytometry following *HSPA9* knockdown by siRNA in human CD34+ cells (N = 3 for each siRNA). (c) Representative plot of erythroid maturation following *HSPA9* knockdown by siRNA measured by flow cytometry (N = 3 for each siRNA). (d) Quantification of CD71+ cells following *HSPA9* knockdown by siRNA measured by flow cytometry (N = 3 for each siRNA). All error bars represent SD, N = 3 technical replicates, representative of two independent experiments. ***P < 0.001. Abbreviations used: SD, standard deviation; siRNA, small interfering RNA.

Del(5q) MDS patients present with anemia and cytopenias, and increased apoptosis in their erythroid cells. To determine if *HSPA9*/mortalin is involved in these clinical findings, we measured the growth, apoptosis, and erythroid maturation of human CD34+ cells after *HSPA9* knockdown by siRNA. After transfecting *HSPA9* and control siRNA into cells, we counted the cell number for five consecutive days. We found that starting from day 4, compared to control siRNA, *HSPA9* siRNA significantly inhibited cell growth (*P* < 0.001) (Figure 2(a)). *HSPA9* knockdown also significantly increased cell apoptosis in human CD34+ cells, indicated by the increased percentage of annexin-V positive cells (16.10 ± 1.50 vs. 6.20 ± 1.12, *P* < 0.001) (Figure 2(b)). Flow cytometry was performed to measure erythroid maturation using CD71 as a surrogate marker. The percentage of immature erythroid cells (CD71−, glycophorin A−) is significantly higher in *HSPA9* siRNA-treated cells compared to control siRNA-treated cells (68.71 ± 2.1 vs. 33.30 ± 3.71, respectively, *P* < 0.001) (Figure 2(c)). In addition, *HSPA9* siRNA significantly inhibited the percentage of CD71+ cells compared to control siRNA (62.20 ± 2.51 vs. 30.11 ± 1.80, respectively, *P* < 0.001, Figure 2(d)), suggesting *HSPA9* knockdown inhibited erythroid maturation of CD34+ cells.

### Pharmacologic inhibition of *HSPA9*/mortalin increased p53 expression and inhibited erythroid maturation in human CD34 + hematopoietic progenitor cells

**Fig. 3:**
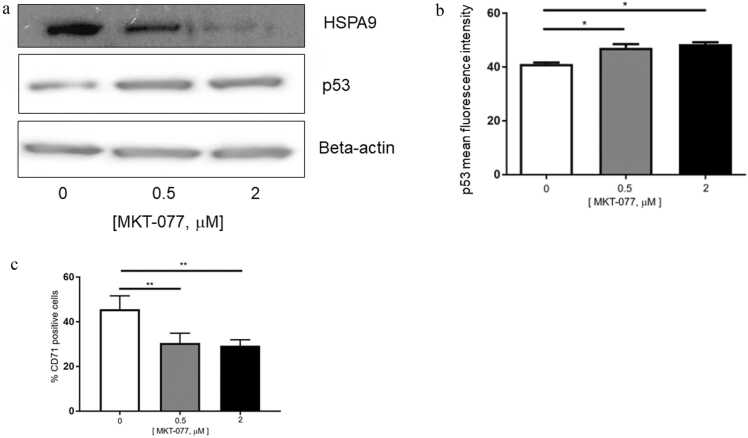
*HSPA9*/mortalin inhibitor MKT-077 increases p53 expression and inhibits erythroid maturation in human CD34+ hematopoietic progenitor cells. (a) Expression of *HSPA9*/mortalin and p53 in human CD34+ cells following MKT-077 treatment for 5 days measured by western blotting. Beta-actin was used as the loading control. The blots shown are a presentation of two independent experiments. (b) Mean fluorescence intensity of p53 following MKT-077 treatment measured by flow cytometry (N = 3). (c) Quantification of CD71+ cells following *HSPA9* knockdown by siRNA measured by flow cytometry (N = 3). All error bars represent SD, N = 3 technical replicates, representative of two independent experiments. *P < 0.05, **P < 0.01. Abbreviations used: SD, standard deviation; siRNA, small interfering RNA.

Next, we studied the effects of pharmacologic inhibition of *HSPA9*/mortalin on p53 expression and erythroid maturation in CD34+ cells using MKT-077, a small molecule inhibitor of HSP70 protein family members including *HSPA9*/mortalin.^28^ Human CD34+ cells were treated with two concentrations (0.5 and 2 μM) of MKT-077 in erythroid differentiation media for 5 days. Following 5 days of treatment, mortalin expression was reduced in a dose-dependent manner measured by western blotting (Figure 3(a)). Similar to *HSPA9* siRNA, MKT-077 also increased the p53 expression levels measured by western blotting (Figure 3(a)) and flow cytometry (*P* < 0.05) (Figure 3(b)). In addition, MKT-077 treatment significantly inhibited the percentage of CD71+ cells (*P* < 0.01, Figure 3(c)), suggesting pharmacological inhibition of *HSPA9*/mortalin repressed erythroid maturation in CD34+ cells.

### Knockdown of *HSPA9* by shRNA inhibited erythroid maturation in human CD34+ hematopoietic progenitor cells

**Fig. 4:**
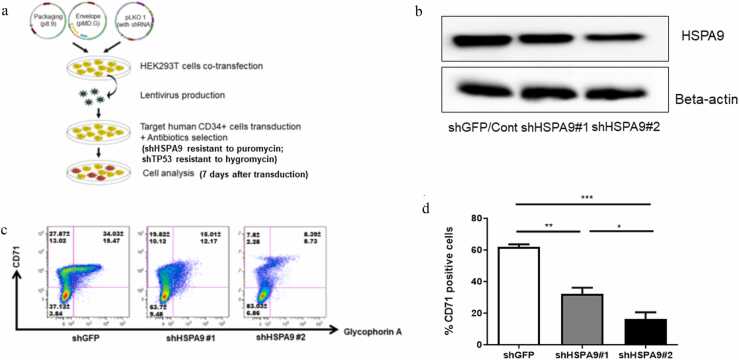
*HSPA9* Knockdown by shRNA inhibits erythroid maturation in human CD34+ hematopoietic progenitor cells. (a) Strategy and flowchart of lentiviral shRNA production and transduction. (b) Expression of *HSPA9*/mortalin after knockdown by shRNA (shGFP/control, shHSPA9#1, or shHSPA9#2) in human CD34+ cells measured by western blotting. Beta-actin was used as the loading control. The blots shown are a presentation of two independent experiments. (c) Representative plot of erythroid maturation following *HSPA9* knockdown by shRNA measured by flow cytometry (N = 3 for each shRNA). (d) Quantification of CD71+ cells following *HSPA9* knockdown by shRNA measured by flow cytometry (N = 3 for each shRNA). All error bars represent SD, N = 3 technical replicates, representative of two independent experiments. *P < 0.05, **P < 0.01, ***P < 0.001. Abbreviations used: SD, standard deviation; shGFP, short hairpin targeting GFP; shRNA, short hairpin RNA.

In order to further test the effects of *HSPA9* knockdown in human CD34+ hematopoietic progenitor cells, we constructed two previously reported short hairpin RNAs (shRNAs) targeting the human *HSPA9* gene [shHSPA9#1 (sh433 in prior study) and shHSPA9#2 (sh960 in prior study)].^27^ The strategy and flowchart of lentiviral shRNA production and transduction are illustrated in Figure 4(a). The shRNAs targeting *HSPA9* (shHSPA9#1 and shHSPA9#2) are resistant to the antibiotic puromycin. Cells were incubated in erythroid differentiation media with shRNAs for 7 days. We measured the *HSPA9*/mortalin level by western blotting, and both shHSPA9#1 and shHSPA9#2 reduced the *HSPA9* protein level to approximately 50% and 20%, respectively, compared to the control shRNA targeting green fluorescent protein (GFP) (Figure 4(b)). Consistent with the effects of inhibiting *HSPA9* by siRNA and MKT-077, knockdown of *HSPA9* by shRNA showed significant inhibition of erythroid maturation in human CD34+ cells cultured in erythroid differentiation media compared to control shRNA, indicated by the reduced percentage of CD71+ cells (Figures 4(c) and (d)).

### *TP53* inhibition reversed erythroid maturation disruption by *HSPA9* inhibition in human CD34+ hematopoietic progenitor cells

**Fig. 5:**
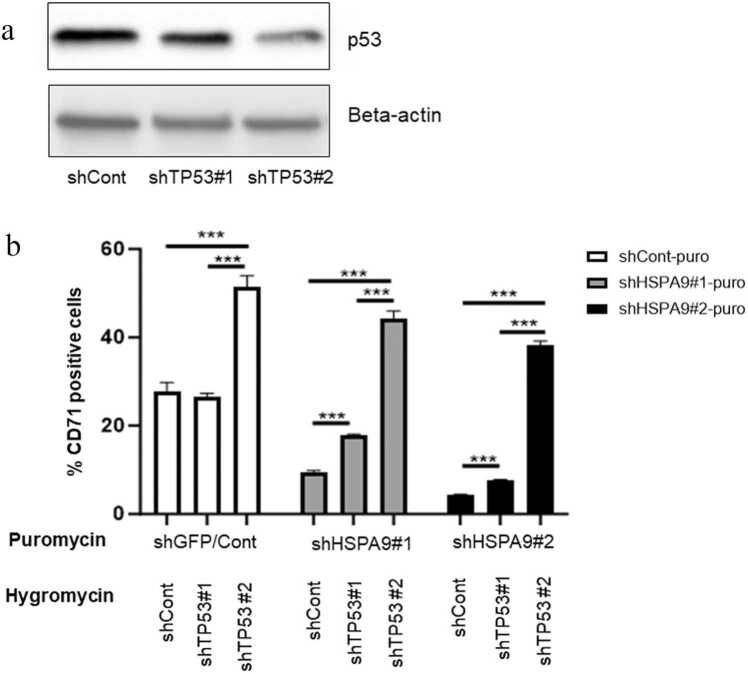
*TP53* inhibition partially reverses erythroid maturation disrupted by *HSPA9* inhibition in human CD34+ hematopoietic progenitor cells. (a) Expression of p53 after knockdown by shRNAs in human CD34+ cells measured by western blotting. Beta-actin was used as the loading control. The blots shown are a presentation of two independent experiments. (b) CD34+ cells were cotransduced with lentiviral constructs carrying an shRNA targeting *TP53* with a hygromycin resistance gene (shGFP/control, shTP53#1, or shTP53#2) and an shRNA targeting *HSPA9* with a puromycin resistance gene (shGFP/control, shHSPA9#1, or shHSPA9#2). Cells were grown in the presence of both hygromycin and puromycin and the fold change in the percentage of CD71+ cells was measured by flow cytometry (N = 3 technical replicates, representative of 2 independent experiments). *P < 0.05, **P < 0.01, ***P < 0.001. Abbreviation used: GFP, green fluorescent protein; shGFP, short hairpin targeting GFP; shRNA, short hairpin RNA.

In order to explore the molecular mechanism of *HSPA9* regulating erythroid maturation, especially whether it is *TP53*-dependent or not, we constructed previously reported shRNAs targeting the human *TP53* gene [shTP53#1 (#4 in prior study) and shTP53#2 (#3 in the prior study)].^27^ The shRNAs targeting *TP53* (shTP53) are resistant to antibiotic hygromycin. shTP53#1 and shTP53#2 were able to decrease p53 protein levels by ∼50% and 80%, respectively, compared to control short hairpin targeting GFP (shGFP) measured by western blotting (Figure 5(a)). Since we previously reported that knockdown of *HSPA9* induces *TP53*-dependent apoptosis in human CD34+ cells, next, we tested whether erythroid maturation inhibition induced by *HSPA9* knockdown is also *TP53*-dependent or not. We cotransduced lentiviruses containing shHSPA9 and shTP53 into human CD34+ cells, followed by double antibiotic selection with puromycin and hygromycin, respectively. Cells were incubated in erythroid differentiation media with shRNAs for 7 days. The results of the experiment are shown in Figure 5, and additional statistical analyses are listed in Table 2. In cultures transduced with the shCont-hygrogmycin virus (i.e., shTP53 control virus), there was the expected reduction in CD71+ cells in shHSPA9#1 and shHSPA9#2 cultures compared to shCont-puromycin cultures (i.e., shHSPA9 control virus) (Figure 5(b), columns 4 and 7 compared to column 1, respectively). There was an increase in the percent of CD71+ cells in shHSPA9#1 and shHSPA9#2 cultures treated with shTP53#1 compared to shCont-hygromycin cultures (i.e., shTP53 control virus), but not in sh-Cont-puromycin cultures (i.e., shHSPA9 control virus) (Figure 5(b), columns 5 and 8 compared to column 2, respectively). There was a similar increase in the percent of CD71+ cells in shCont-puromycin, shHSPA9#1, and shHSPA9#2 cultures treated with shTP53#2 (Figure 5(b), columns 6 and 9 compared to column 3, respectively). These data suggest that erythroid maturation inhibition by *HSPA9* knockdown is partially mediated through a *TP53* mechanism. We also noticed that shTP53#2 affects control cells similar to shHSPA9 cells (Figure 5(b), column 3 compared to column 1). This is probably due to that *TP53* is necessary for normal erythroid maturation at some level, and when its level is reduced to below 50% levels, it impacts normal erythroid maturation and actually induces it more.

**Table 1:** Short hairpin RNA sequences.

shRNA	Vector	Forward (5′-sense)	Loop	Reverse (3′-antisense)
GFP (Control)	pLK0.1	GCAAGCTGACCCTGAAGTTCA	CTCGAG	TGAACTTCAGGGTCAGCTTGC
HSPA9#1	pLK0.1	GCTGTCACCAACCCAAACAAT	CTCGAG	ATTGTTTGGGTTGGTGACAGC
HSPA9#2	pLK0.1	GCACATTGTGAAGGAGTTCAA	CTCGAG	TTGAACTCCTTCACAATGTGC
TP53#1	pLK0.1	TCAGACCTATGGAAACTACTT	CTCGAG	ATTCTCTTCCTCTGTGCGCCG
TP53#2	pLK0.1	GTCCAGATGAAGCTCCCAGAA	CTCGAG	ATGTAGTTGTAGTGGATGGTG

Abbreviation used: GFP, green fluorescent protein; shRNA, short hairpin RNA.

The sequences of shRNAs used in the study are listed, including shRNAs targeting GFP (control), *HSPA9*, and *TP53*.

**Table 2:** Statistical analysis results of Figure 5**.**

Column A (column # in Figure 5)	Column B (column # in Figure 5)	Statistical analysis results (column A vs. column B)
*Statistics already labeled in*Figure 5**:**		
shCont-hygroshCont-puro (column #1)	shTP53#1-hygroshCont-puro (column #2)	*P* > 0.05
shCont-hygro shCont-puro (column #1)	shTP53#2-hygro shCont-puro (column #3)	*P* < 0.001
shTP53#1-hygro shCont-puro (column #2)	shTP53#2-hygro shCont-puro (column #3)	*P* < 0.001
shCont-hygro shHSPA9#1 (column #4)	shTP53#1-hygro shHSPA9#1-puro (column #5)	*P* < 0.001
shCont-hygro shHSPA9#1 (column #4)	shTP53#2-hygro shHSPA9#1-puro (column #6)	*P* < 0.001
shCont-hygro shHSPA9#1 (column #5)	shTP53#2-hygro shHSPA9#1-puro (column #6)	*P* < 0.001
shCont-hygro shHSPA9#1-puro (column #7)	shTP53#1-hygro shHSPA9#2-puro (column #8)	*P* < 0.001
shCont-hygro shHSPA9#1-puro (column #7)	shTP53#2-hygro shHSPA9#2-puro (column #9)	*P* < 0.001
shTP53#1-hygro shHSPA9#2-puro (column #8)	shTP53#2-hygro shHSPA9#2-puro (column #9)	*P* < 0.001

*Statistics not labeled in*Figure 5**:**		
shCont-hygro shCont-puro (column #1)	shCont-hygro shHSPA9#1-puro (column #4)	*P* < 0.001
shCont-hygro shCont-puro (column #1)	shCont-hygro shHSPA9#2-puro (column #7)	*P* < 0.001
shCont-hygro shHSPA9#1-puro (column #4)	shCont-hygro shHSPA9#2-puro (column #7)	*P* < 0.001
shTP53#1-hygro shCont-puro (column #2)	shTP53#1-hygro shHSPA9#1-puro (column #5)	*P* < 0.001
shTP53#1-hygro shCont-puro (column #2)	shTP53#1-hygro shHSPA9#2-puro (column #8)	*P* < 0.001
shTP53#1-hygro shHSPA9#1-puro (column #5)	shTP53#1-hygro shHSPA9#2-puro (column #8)	*P* < 0.001
shTP53#2-hygro shCont-puro (column #3)	shTP53#2-hygro shHSPA9#1-puro (column #6)	*P* < 0.05
shTP53#2-hygro shCont-puro (column #3)	shTP53#2-hygro shHSPA9#2-puro (column #9)	*P* < 0.001
shTP53#2-hygro shHSPA9#1 (column #6)	shTP53#2-hygro shHSPA9#2-puro (column #9)	*P* < 0.01

Each group or column presented in Figure 5 was compared using one-way ANOVA with a Tukey post-test.

## Discussion

Mortalin, encoded by the *HSPA9* gene, is a highly conserved heat shock chaperone belonging to the HSP70 family. It is predominantly presented in the mitochondria but is also found in other subcellular compartments including the plasma membrane, endoplasmic reticulum, and cytosol.^29^ *HSPA9*/mortalin is critical in regulating a variety of cell physiological functions such as response to cell stress, control of cell proliferation, and inhibition/prevention of apoptosis,^30^ which may explain our observation that *Hspa9* homozygous deletion mice are embryonic lethal.^23^

Anemia, one of the significant clinical findings presented in MDS patients, is commonly treated with blood transfusions and erythropoietin.^31^ Bone marrow samples from del(5q) MDS patients display increased apoptosis associated with increased expression of *TP53* and its target genes in erythroid cells.^32^, ^33^ During the early stage of erythropoiesis, hematopoietic stem cells sequentially give rise to the common myeloid progenitor, megakaryocyte-erythrocyte progenitor, burst-forming unit-erythroid, and colony-forming unit-erythroid cells.^34^ Our present study addresses the effects of *HSPA9* expression levels on erythroid maturation in human CD34+ hematopoietic progenitor cells. We observed that knockdown of the *HSPA9* gene located on the proximal chromosome 5 CDR could inhibit erythroid maturation in CD34+ cells cultured in erythroid differentiation media, consistent with our prior results.^35^ In addition, our previous studies demonstrated that *HSPA9* knockdown induces apoptosis in human CD34+ cells, through a *TP53*-dependent mechanism.^27^ In this report, we address whether inhibition of erythroid maturation by *HSPA9* knockdown is *TP53*-dependent or not. We cotransduced lentiviruses containing shRNA targeting *HSPA9* and *TP53* genes into human CD34+ cells. We observed that *HSPA9*/mortalin inhibition disrupted erythroid maturation by decreasing the percentage of CD71+ cells in a *HSPA9* level-dependent manner. In addition, *TP53* shRNAs could partially reverse the erythroid inhibition caused by *HSPA9* shRNAs, indicating that erythroid maturation regulated by *HSPA9* knockdown is also *TP53*-dependent.

*HSPA9*/mortalin is reduced by ∼50% in del(5q) MDS cells, consistent with haploinsufficient levels.^20^ In this study we show that the proximal del(5q) candidate gene *HSPA9* regulates erythroid maturation in human CD34+ cells, suggesting *HSPA9*/mortalin may be a potential target to treat anemia in del(5q) MDS patients by reactivating its expression from the residual wild-type nondeleted allele. Similarly, the ribosomal protein gene *RPS14* is also a del(5q) candidate gene located on the distal chromosome 5 CDR. The Ebert lab reported that haploinsufficiency of *RPS14* causes activation of p53 in human erythroid progenitor cells and a block in erythroid differentiation, as well as inducing apoptosis in a mouse model. They also reported that mice with *Rps14* haploinsufficiency in hematopoietic cells developed a progressive anemia associated with the induction of a p53-dependent erythroid differentiation defect in late-stage erythroblasts.^32^, ^36^ Huang *et al*.^37^ reported that the deficiency of *SF3B1*, a core component of the splicing machinery, also impairs human erythropoiesis *via* activation of p53 in human CD34+ cells. Collectively, these and our studies confirm that insufficiency of specific MDS-associated genes, including *HSPA9,* could disrupt erythroid maturation *via TP53* -dependent mechanisms, providing insights into ineffective erythropoiesis in MDS patients.

Studies have shown that p53 activation during ribosome biogenesis regulates normal erythroid differentiation using human CD34+ cells and mouse models.^38^ It has also been confirmed by multiple studies and in multiple cancer types that *HSPA9*/mortalin interacts with p53 and the regulation of apoptosis by *HSPA9*/mortalin is *TP53*-dependent.^27^, ^39^, ^40^ However, the precise mechanism of how *HSPA9*/mortalin regulates erythroid maturation, especially in del(5q) or 5q− syndrome, is unclear. As discussed above, some studies showed haploinsufficiency of certain del(5q) genes such as *RPS14* led to p53 activation and underlies the anemia in the 5q− syndrome. For the *HSPA9* gene, a clinical study^41^ and a study using yeast^42^ identified that *HSPA9* mutations may contribute to congenital sideroblastic anemia, implicating a role of *HSPA9* in erythroid maturation. In this study, we found that *HSPA9*/mortalin inhibition disrupts erythroid maturation dependent on *TP53* using human CD34+ hematopoietic progenitor cells, consistent with the report by Caceres *et al*.^43^ that *TP53* suppression promotes erythropoiesis in del(5q) MDS. Collectively, our results suggest that the increased apoptosis and reduced erythroid maturation observed in del(5a)-associated MDS is *TP53*-dependent. Thus, *HSPA9*/mortalin may also be a potential target to treat anemia in del(5q) MDS patients, although the simultaneous loss of multiple genes on del(5q) likely contributes to the complex phenotypes observed in MDS. We believe that inhibition of erythroid maturation as indicated by decreasing the CD71 surface marker by *HSPA9* knockdown is partially due to increased apoptosis through *TP53*, but there are additional mechanisms. In our study, when we measured the CD71 level by flow cytometry, we gated and measured in most live cells rather than apoptotic cells. This indicated that decreased CD71 is probably due to the impact of *HSPA9* knockdown on erythroid differentiation. The mechanism of how *HSPA9*/mortalin regulates erythrogenesis has been explored. For example, Shan *et al*.^44^ reported that *HSPA9*/mortalin enhances the synthesis of mitochondrial iron–sulfur cluster in yeast, which is required for heme synthesis and erythroid differentiation. Yamamoto *et al*.^45^ reported that mortalin cooperates with the inner mitochondrial translocase complex to facilitate the translocation of mitochondrial matrix proteins that are essential for mitochondrial function and cell viability in yeast. Therefore, along with the studies we have done, it is conceivable that depletion of *HSPA9* could lead to increased mitochondrial dysfunction and activation of proapoptotic factors that induce hematopoietic progenitor cell death and maturation dysfunction. Intriguingly, we also showed that compared to progenitors of other lineages of hematopoiesis, a greater reduction of burst-forming unit-erythroid progenitors was observed when *HSPA9* was knocked down in mice, suggesting that *HSPA9* possibly plays an additional role(s) in maintaining the erythroid progenitor cell niche.^35^ However, it is unclear whether the decreased erythroid maturation marker CD71 caused by *HSPA9* knockdown or chemical MKT-077 is due to higher levels of apoptosis in CD71 or not. First, from clinical aspects, del(5q) MDS patients are characterized by ineffective hematopoiesis (erythroid unable to mature) and peripheral blood cytopenia (due to increased apoptosis of bone marrow cells), both contributing to anemia in patients. Thus, it is hard to differentiate whether the clinical symptom of anemia is caused by apoptosis or erythroid maturation inhibition separately. Second, from basic science aspects, our studies have demonstrated that *HSPA9* regulates both apoptosis^27^ and erythroid maturation (this study) through a *TP53*-dependent mechanism. Thus, the precise relationship between apoptosis and erythroid maturation regulated by *HSPA9* is well worth to be further investigated.

## Methods

### Cells and reagents

Granulocyte colony-stimulating factor mobilized human peripheral blood CD34+ hematopoietic progenitor cells were obtained from the Fred Hutchinson Cancer Research Center (Seattle, WA, USA) (purity of CD34+ cells was higher than 90%). The CD34+ progenitor cells were primed overnight in media containing X-VIVO 15 media (Lonza Inc., Walkersville, MD, USA), human cytokines (PeproTech, Inc., Cranbury, NJ, USA) including 50 ng/mL of stem cell factor, 50 ng/mL of Fms-related tyrosine kinase 3 ligand, 50 ng/mL of thrombopoietin, 50 ng/mL of interleukin 3 (IL-3), and L-glutamine (Thermo Fisher Scientific). The cells were subsequently cultured for 5 days in erythroid differentiation media containing serum-free expansion medium (STEMCell Technologies, Inc., Vancouver, Canada), human cytokines (PeproTech, Inc.) including 25 ng/mL of stem cell factor, 10 ng/mL of IL-3, 10 ng/mL of IL-6, 3 units/mL of erythropoietin, 100 U/mL of penicillin/streptomycin, and 2 mM of L-glutamine (Sigma-Aldrich Inc.).^27^ HEK293T cells (ATCC Inc., Manassas, VA, USA) were cultured in Dulbecco’s Modified Eagle’s Medium (ATCC Inc.) containing 10% fetal bovine serum (ATCC Inc.), and 2 mM L-glutamine (Sigma-Aldrich Inc.). All the cells were cultured at 37 °C with 5% CO_2_. The chemical MKT-077 was obtained from Sigma-Aldrich Inc. (St Louis, MO, USA).

### siRNA transduction

The procedure of siRNA transduction was described previously.^23^ Briefly, human CD34+ progenitor cells were electroporated with 1000 nmol/L of SMARTpool *HSPA9*-targeting siRNAs (L-004750-00-0005; Dharmacon, Inc., Lafayette, CO, USA) or non-targeting control siRNA (D-001810-10-05, Dharmacon, Inc.) using the Amaxa human CD34+ cell nucleofector kit and Nucleofector Device (Lonza Inc) with the Nucleofector program U-008 following the manufacturer's protocol.

### Cloning of lentiviral shRNA vector

shRNAs targeting the *HSPA9* were cloned into pLKO.1 vector (Addgene Inc., Watertown, MA, USA) carrying the puromycin resistance gene and shRNAs targeting *TP53* were cloned in the same vector carrying the hygromycin resistance gene following the manufacturer’s protocol. shRNA oligonucleotides (oligos) were designed based on the information from the RNA interference (RNAi) Consortium of the Broad Institute and synthesized by Integrated DNA Technologies, Inc. (Coralville, IA, USA). shRNA sequences are listed in Table 1. Briefly, first, for oligo annealing, the oligos were resuspended in ddH2O to a concentration of 20 μM then mixed with 5 μL of forward oligo, 5 μL of reverse oligo, 5 μL of 10× New England Biolabs (NEB) buffer 2 (New England Biolabs, Inc., Ipswich, MA, USA), and 35 μL of ddH2O. The 40 μL mixture was incubated in an Eppendorf Mastercycler thermocycler (Hamburg, Germany) at 95 °C for 5 min, then 70 °C for 5 min, followed by lowering the temperature by 5 °C at every 5 min interval, until room temperature and held. Second, for pLKO.1 cloning vector digestion, the vector was digested with restriction enzymes (New England Biolabs, Inc.) *Age*I and *Eco*RI follow the manufacturer’s protocol. The digested vectors were purified using the QIAquick gel extraction kit (QIAGEN, Inc., Hilden, Germany). For vector ligating and transforming, a total of 20 μL final volume of mixture [including 2 μL of the annealed oligo, 20 ng of digested pLKO.1, 2 μL 10× NEB T4 DNA ligase buffer (New England Biolabs, Inc.), 1 μL NEB T4 DNA ligase (New England Biolabs, Inc.), and ddH2O] was incubated at 16 °C for 20 h. Next, 2 μL of ligation mix was transformed into 25 μL DH5-alpha *E. coli* competent cells (Invitrogen, Inc., Carlsbad, CA) following the manufacturer’s protocol. The transformations were spread on ampicillin-selective plates and incubated overnight at 37 °C. Colonies were picked and cultured in Luria-Bertani (LB) medium containing 100 μg/mL ampicillin with shaking at 250 rpm overnight at 37 °C. On the next day, plasmid DNA was isolated by using the Invitrogen PureLink quick plasmid miniprep kit (Invitrogen, Inc.). The correct positive clones were confirmed by sequencing using the pLKO.1 sequencing primer (5′-CAA GGCTGTTAGAGAGATAATTGGA-3′) at the West Virginia University Genomics Core Facility.

### Lentiviral shRNA production, transduction, and culture

The process of lentiviral shRNA production and transduction is illustrated in Figure 4(a). The pLKO.1 vector containing shRNA and lentiviral vectors were transfected into HEK293T cells using the calcium phosphate transfection method. The p8.9 and pMD.G vectors were used as packaging and envelop vectors, respectively. Vectors were transfected into HEK293T cells using the CalPhos mammalian transfection kit (Takara Bio USA, Inc., San Jose, CA, USA) following the manufacturer's protocol. The lentiviruses presented in the cell culture media were collected and titered prior to transduction into human CD34+ cells. The shRNAs targeting the human *HSPA9* gene are resistant to the antibiotic puromycin, and shRNAs targeting *TP53* (shTP53) are resistant to antibiotic hygromycin respectively. The transduction of lentiviral shRNA into human CD34+ cells and associated culture conditions have been described previously.^27^, ^35^ Briefly, CD34+ progenitor cells were primed in X-VIVO 15 media with human cytokines overnight before lentiviral transduction. Cells were spinoculated in the presence of polybrene (5 μg/mL) and incubated overnight at 37 °C in 5% CO_2_. Cells were washed and incubated in erythroid differentiation media for 7 days.

### Western blotting

The Western blotting assay was described previously.^27^, ^46^ Briefly, cell lysates were prepared in radioimmunoprecipitation assay (RIPA) buffer, and protein samples were loaded onto an sodium dodecyl sulfate (SDS)-polyacrylamide gel, separated by electrophoresis, and subsequently transferred to a polyvinylidene fluoride (PVDF) membrane. Membranes were blocked with 5% milk in 1× tris-buffered saline (TBS) containing 0.05% (v/v) Tween-20 for 4 h at room temperature and washed seven times with 1× TBS and 1× tris-buffered saline with 0.1% Tween 20 detergent (TBST) alternatively. Membranes were then incubated with the primary antibody overnight at 4 °C followed by incubation with the secondary antibody at room temperature for 1 h. Pierce supersignal chemiluminescent substrates were used, and images were captured by using the G:BOX Chemi XT4 gel documentation system (Syngene Inc., Frederick, MD, USA). The following antibodies were used: *TP53*/p53 (SC-126; Santa Cruz Biotechnology, Inc., Dallas, TX, USA), *HSPA9*/mortalin (MA3-028; Affinity BioReagents, Inc., Denver, CO, USA), and beta-actin (A5441, Sigma-Aldrich, Inc.).

### Flow cytometry

Cell apoptosis and p53 levels were measured and analyzed using the BD Accuri C6 flow cytometry apparatus (BD Biosciences, Inc., San Jose, CA, USA). Cell apoptosis was measured using the PE Annexin V apoptosis detection kit (BD Biosciences, Inc.) following the manufacturer's protocol, and the percentage of apoptotic cells was detected and analyzed by flow cytometry, as described previously.^27^ To measure the level of intracellular p53, intracellular analysis by flow cytometry was used as described previously.^27^, ^32^ Briefly, CD34+ cells were fixed with 2% paraformaldehyde for 15 min at 37 °C and permeabilized with ice-cold methanol for 30 min at −20 °C. Cells were then incubated for 1 h with the p53 antibody followed by analysis using flow cytometry. The following antibodies were used: CD71 (fluorescein isothiocyanate (FITC), 11-0719-42, eBioscience/Thermo Scientific, Inc., Waltham, MA, USA), glycophorin A (PE, 12-9987-80, eBioscience, Inc.), and p53 (Alexa647, 2533S, Cell Signaling Technology, Inc., Danvers, MA, USA).

### Statistics

The statistical significance of the data between the two groups was analyzed by the Student t-test (GraphPad Prism 10). The statistical significance of the data with more than two groups was analyzed by one-way analysis of variance (ANOVA) with a Tukey post-test (GraphPad Prism 10). Significance levels were set at *P* < 0.05 (labeled *), *P* < 0.01 (labeled **), and *P* < 0.001 (labeled ***).

## Significance statement

MDS is the most common adult myeloid blood cancer in the United States. Typical symptoms of MDS patients includes fatigue and pallor, which are associated with anemia. Some MDS patients harbor del(5q), with haploinsufficiency for a set of genes including *HSPA9*. We showed that inhibition of *HSPA9* disrupts erythroid maturation in human CD34+ hematopoietic progenitor cells, through a *TP53*-dependent mechanism. Our findings not only indicate that reduced levels of *HSPA9* may contribute to anemia observed in del(5q)-associated MDS patients, but also provide new insights into potential mechanisms of anemia.

## Author contribution

Christopher Butler: Performed research, analyzed and interpreted data; Morgan Dunmire: Performed research, manuscript writing; Jaebok Choi: Analyzed and interpreted data, manuscript writing; Gabor Szalai: Performed research, analyzed and interpreted data, manuscript writing; Anissa Johnson: Performed research, manuscript writing; Wei Lei: Analyzed and interpreted data, manuscript writing; Xin Chen: Analyzed and interpreted data, manuscript writing; Liang Liu: Analyzed and interpreted data; Wei Li: Analyzed and interpreted data, manuscript writing; Matthew J. Walter: Financial support, designed research, performed research, analyzed and interpreted data, manuscript writing; Tuoen Liu: Financial support, designed research, performed research, analyzed and interpreted data, manuscript writing.

## Declarations of interest

The authors declare that they have no known competing financial interests or personal relationships that could have appeared to influence the work reported in this paper.

## Data Availability

Data will be made available on request. Manuscript data (Original data) (google drive)
